# The function of serine/threonine-specific protein kinases in B cells

**DOI:** 10.3389/fimmu.2024.1459527

**Published:** 2024-10-09

**Authors:** Zhennan Han, Kamel Benlagha, Pamela Lee, Chan-Sik Park, Alexander Filatov, Maria G. Byazrova, Heather Miller, Lu Yang, Chaohong Liu

**Affiliations:** ^1^ Department of Pathogen Biology, School of Basic Medicine, Tongji Medical College and State Key Laboratory for Diagnosis and Treatment of Severe Zoonotic Infectious Diseases, Huazhong University of Science and Technology, Wuhan, Hubei, China; ^2^ Université de Paris, Institut de Recherche Saint-Louis, EMiLy, Paris, France; ^3^ Department of Paediatrics and Adolescent Medicine, Li Ka Shing Faculty of Medicine, The University of Hong Kong, Hong Kong, Hong Kong SAR, China; ^4^ Department of Pathology, Asan Medical Center, University of Ulsan College of Medicine, Seoul, Republic of Korea; ^5^ Laboratory of Immunochemistry, National Research Center Institute of Immunology, Federal Medical Biological Agency of Russia, Moscow, Russia; ^6^ Cytek Biosciences, R&D Clinical Reagents, Fremont, CA, United States

**Keywords:** STK family protein, B cell, cell differentiation, cell proliferation, B cell activation

## Abstract

The serine/threonine-specific protein kinases (STKs) are important for cell survival, proliferation, differentiation, and apoptosis. In B cells, these kinases play indispensable roles in regulating important cellular functions. Multiple studies on human and other animal cells have shown that multiple STKs are involved in different stages of B cell development and antibody production. However, how STKs affect B cell development and function is still not completely understood. Considering that B cells are clinically important in immunity and diseases, our understanding of STKs’ roles in B cells is in great need of investigation with current technologies. Investigating serine/threonine kinases will not only deepen our insight into B cell-related disorders but also facilitate the identification of more effective drug targets for conditions like lymphoma and systemic lupus erythematosus.

## Background

Serine/threonine-specific protein kinases (STKs) are a family of kinases that phosphorylate the hydroxyl (OH) groups on serine or threonine residues of proteins. This family of kinases, including Akt, MAPK, and Raf are linked to cancerogenesis and are mostly intracellular kinases (1).

STKs are important regulators of cell proliferation, cell differentiation, and apoptosis. Cell proliferation and development are crucial for B cell function. Therefore, it is necessary to study the roles of STKs in B cells in order to fully understand B cell immune responses. Also, due to their role in B cell development, understanding their involvement in the genesis of lymphoma could facilitate the discovery of new targets for chemotherapies.

From pluripotent stem cells to antibody-secreting B cells, B cells undergo a series of well-designed stages for development including proliferation, differentiation, anergy, and cell death (2). STKs are involved in the entirety of B cell development and function, with some of these STKs being indispensable, like Raf and ERKs. After stimulation of B cell receptors (BCRs), the STKs, PKC and RasMAPK, are activated and promote downstream signaling cascades associated with B cell development, such as MAPK/ERK pathways and p38 MAPK signaling pathways (3). However, this is just a simplified example of the role of STKs in B cell development. This review will provide a summary of the roles in B cell development and function of the most extensively studied intracellular STKs. Casein kinase 2 has a crucial role in both normal and malignant hematopoiesis, it is also an important regulator of the differentiation from transitional B (TrB) cells to marginal zone B (MZB) cells. Protein kinase A is indispensable for Somatic hypermutation (SHM) and class-switch recombination (CSR). Protein kinase B (AKT) closely relates to B cell proliferation, survival, growth, and metabolism. PKC has different roles in B cell development as well as B cell function. Raf kinase interacts with MAPK thus affecting B cell development and function. CaMK regulates B cell proliferation and survival via AKT/mTOR signaling. This signaling is also found to be involved in B cell autophagy regulation. IRAK deficient B cells show abnormalities in both numbers and function, indicating its important role in B cells.

## Casein kinase 2

Casein kinase 2 (CK2) belongs to the eukaryotic protein kinase (EPK) superfamily (1). However, unlike the typical EPKs that only utilize ATP, CK2 utilizes both ATP and GTP.

Within tissues, CK2 exists in the form of a heterotetrameric holoenzyme with α_2_β_2_-stoichiometry (4, 5), α and α’ being the catalytic subunits and β being the regulatory subunits (6). But CK2 has different forms in different animals. For example, in insect cells, scientists found CK2β-free CK2α. Also, distinct isoenzyme forms of the catalytic subunit of CK2 exist in many organisms (7). The CK2 knockout or CK2 overexpression shows significant changes in hematopoiesis and embryonic development. NF-κB, JAK/STAT, and PI3K/AKT/mTOR signaling pathways are affected by the change in CK2 expression.

In hematopoietic cells, CK2 regulates signaling pathways and transcriptional factors, such as P65/RelA (8). In B cell acute lymphoblastic leukemia and diffuse large B cell lymphomas, CK2 is overexpressed and overactivated, which causes unregulated activation of NF-κB, JAK/STAT, and PI3K/AKT/mTOR signaling pathways.

Wei et al. demonstrated that CK2α is an important regulator involved in the differentiation of transitional B (TrB) cells into marginal zone B (MZB) cells (9). In their experiment, they found that stimulated B cells had activated CK2 and elevated CK2 expression. When CK2α was knocked out, BCR signaling was reduced and Notch2 signaling was enhanced, leading to an abnormal increase in MZB cells. In CK2α knocked-out mice, BCR-related phosphorylated intermediates such as CD79a, Syk, BTK, PLCg 2, and ERK1/2 have decreased levels of phosphorylation, while Heyl, Hes5, and Dtx1, which related to Notch signaling pathways, have increased expression levels. This proved that CK2α is essential for BCR signaling and negatively regulating Notch2-signaling pathways. However, the mechanism of CK2’s regulation in protein expression remains a topic of ongoing investigation.

Quotti Tubi et al. examine the role of CK2β, a regulatory subunit, in B cells (8). A B cell-specific CK2β knockout model revealed a notable decline in B cell percentage and a reduction in peripheral blood B cell counts. In the spleen, they observed a reduction in the percentage and absolute number of FO B cells and an increase in MZ B cell percentage and absolute number which is a result of the increase in Notch 2 activation. The knockout of CK2β also impairs BCR signaling, GC B cell class switch, and plasmablast generation. By investigating the signaling downstream of TLR, IL-4R, and CD40, which are crucial for the maturation of B cells, Quotti Tubi et al. believe that CK2β positively controls SHM and CSR in GC B cells. They also demonstrated that CK2β supports the selection of B cells expressing high-affinity Ab variants, which is driven by antigen.

Studies on gene-targeted mice revealed that CK2α and β are indispensable for survival, however, the isoform, CK2α’, is dispensable. CK2α knockout mice die *in utero* at mid-gestation and have abnormalities of the neural tube and heart. The histological examination of the heart showed abnormal development of the four chambers and of the endocardium endothelium (10). One study showed that CK2β knockout mice also died *in utero* at early gestation because of a cell autonomous defect (11). CK2α’ knockout mice survive and develop normally with no defects in hematopoietic cells.

Collectively, these studies indicate that the CK2 holoenzyme, as well as the CK2α and CK2β subunits, has an indispensable role in B cell differentiation and function.

## Protein kinase A

Cyclic AMP-dependent protein kinase A (PKA) is a tetrameric holoenzyme, which consists of two regulatory subunits and two catalytic subunits, in its inactive state (12). The activation of PKA requires the activation of G-protein-coupled receptors (GPCRs) signaling followed by the elevation of cAMP. A-kinase anchoring protein (AKAP) is another protein needed for the regulation of PKA activation. AKAP binds to the regulatory subunits and other molecules, bringing PKA closer to its target protein (13).

cAMP is found to be crucial for immune cell function. cAMP is a necessary component in antigen-stimulated activation induction meanwhile limits the activation via negatively regulating the BCR signaling. In B cells, the induction of B cell proliferation and antibody production requires cAMP as an essential second messenger (14).

Somatic hypermutation (SHM) and class-switch recombination (CSR) are critical for diversifying the Ig genes. Both SHM and CSR need activation-induced cytidine deaminase (AID) (15). AID has the ability to deaminase single-stranded DNA (ssDNA) (16). The AID caused deaminase generates uracil, which triggers the DNA repair events. This DNA repair event ultimately results in the Ig V region mutation in SHM and DNA double strand breaks (DSB) in IgH switch(S) regions during CSR (17). Collectively, PKA, which acts as an AID kinase, is naturally thought as a regulator in SHM and CSR in germinal center B cells.

Using co-purification and co-immunoprecipitation, Basu et al. showed that in activated B cells, PKA interacts with AID and that PKA is involved in the post-translational regulation of AID (18). In their mouse experiments, they revealed that PKA phosphorylates the serine 38 residue of AID. Following phosphorylation, activated AID interacts with Replication Protein A (RPA), which promotes CSR.

Pasqualucci et al. proved that PKA is a regulator of AID (19). By using pharmaceutical reagents to inhibit PKA, their team was able to prevent CSR in a murine B-cell lymphoma cell line. Furthermore, they conditionally deleted the inhibitory subunit of PKA and found that CSR was enhanced. Based on the fact that PKA phosphorylates AID in the cytoplasm of B cells, they deduced that PKA is an important regulator of AID function and controls T cell dependent immune responses by PKA-activating signals.

## Protein kinase B

Protein kinase B (AKT) has important roles in multiple cellular processes. In B cells, AKT is known to be critical for the maturation and survival of peripheral B cells (20). Calamito et al. demonstrated that the generation of MZB and B1 cells requires both AKT1 and AKT2. Their studies showed that AKT1 and AKT2 deficient mice have disrupted the development of precursors into MZB and B1 cells. They also found that AKT1 and AKT2 deficient MZB and B1 cells have lower survival rates, which was due to inefficient BCR signaling. Additionally, AKT1 and AKT2 are also needed for the transition of immature T2 B cells into mature B cells.

**Figure 1:**
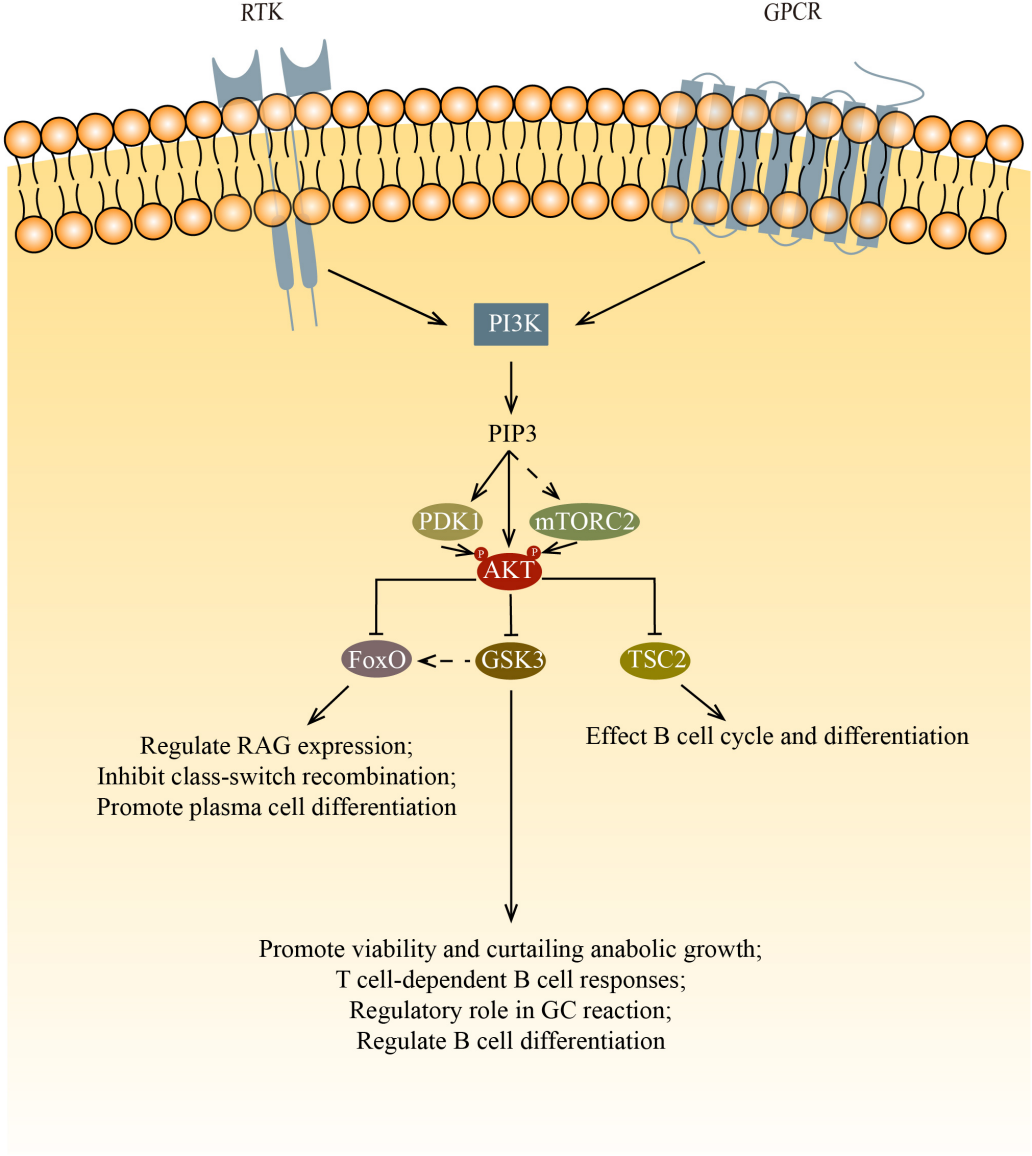
AKT regulates cell survival, proliferation, metabolism, and growth by phosphorylating multiple down-stream substrates. This figure shows the three main targets phosphorylated by AKT: FoxO, GSK3 and TSC2. FoxO inhibits survival and proliferation while promoting metabolism. In B cells, FoxO affects B cell development and differentiation via regulating RAG expression, inhibiting class-switch recombination and promoting plasma cell differentiation. GSK3 inhibits survival, proliferation, and metabolism after being phosphorylated by AKT. In B cells, GSK3 affects B cell survival, proliferation and function. After the inactivation of GSK3, FoxO1 relocalize in nucleus, this increase of FoxO1 initiate the transition from LZ to DZ. By inhibiting the mTOR signaling pathway via TSC2, AKT affects cell cycle and differentiation.

The PI3K/AKT pathway is downstream of the BCR and has multiple roles in the development and function of B cells. Activated AKT phosphorylates downstream targets, thus promoting cell survival, metabolism, growth, and proliferation (**Figure 1**). AKT has multiple cellular targets that it modulates for controlling key signaling nodes (21).

For normal B cell development, both PDK1 and mTORC2 are needed to phosphorylate AKT at Thr 308 and Ser 473, respectively. Phosphorylation by mTORC2 and PDK1 allows AKT to inactivate Foxo1 and promote proper B cell development (22). Foxo1 regulates Rag expression, which is needed for the differentiation and expansion of both pro-B cells and pre-B cells. Ablating the component of mTORC2 leads to the decreased phosphorylation of AKT in Ser 473 and Foxo1, ultimately results in the failure of downregulation of Rag and il7r expression (23, 24). PDK1 is also important in B cell development and functions. PDK1 deficient mouse B cells have low AKT phosphorylation as well as low Foxo1 phosphorylation (25, 26). In Baracho’s study, the germinal center development was severely impaired in PDK1 KO mature B cells. Venigalla et al. demonstrate that PDK1 regulates the cell cycle, cell survival, and the arrangement of Ig heavy chain, which is one of the processes during the transition of pro-B to pre-B cells. PDK1 is required in IL7 induced AKT activation. The loss of PDK1 has been demonstrated to result in a reduction in the phosphorylation of AKT, furthermore, a decrease in the basal phosphorylation of FoxO. PDK1 also regulates the expression of Bcal2A1 and Pax5 in pre-B cells, thereby regulating the B cell differentiation.

Once phosphorylated, the multiple targets of AKT affect B cell differentiation and function via their own pathways. Among those targets, mTORC1, the glycogen synthase kinase 3 (GSK3), and forkhead box O (FoxO) family transcription factors are of most significance.

Phosphorylated AKT inhibits the mTOR signaling pathway by phosphorylating the tuberous sclerosis complex (TSC1/TSC2). The significance of mTOR in the normal development of B cells is not widely studied. Though rapamycin, which is known to inhibit mTOR pathways, suppresses lymphocyte proliferation, it is still no fully understood how rapamycin blocks B cell cycle and differentiation. Rapamycin was found to impede the kinetics of B cell proliferation and delay B cell activation. However, the use of rapamycin doesn’t lead to cell death. Rapamycin also shows no inhibition in the increase of B cell surface, which indicates B cell have left G0 phase after activation. Therefore, rapamycin was deduced to act after the initial activation events (27). Kay et al. and Aagaard-Tillery et al. both demonstrate that rapamycin acts at late stage of B cell activation (28, 29). Aagaard-Tillery et al. also proved that rapamycin addition leads to a complete block in differentiation of B cell to Antibody-Secreting Cells (ASCs). To date, there are no publications on mTORC1 deficient B cells. However, there is only one describing the results of B cells that lost TSC1 (the negative regulator of mTOR). This study found that the percentage of MZ B cells was reduced and the maturation of B cells was impaired (30). They suspected that it was due to the reduced activity of the upstream PI3K/AKT signaling, which is caused by the elevated mTORC1 activity in transitional B cells via a negative feedback loop.

The GSK3 is also phosphorylated by AKT. The phosphorylated GSK3 can activate multiple cell growth regulators such as c-Myc and cyclin D3 (31, 32). Jellusova et al. showed that GSK3 is essential in the quiescence, homeostasis, and long-term survival of mature B cells as well as the growth and proliferation of GC B cells (33). It was suggested that the promotion of cell growth and survival is due to GSK3’s ability to regulate the metabolism of B cells. In support of this, they found that GSK3 regulates B cell anabolic growth during glucose deprivation. In addition to the necessary cellular processes mentioned above, GSK3 is also involved in B cell activation and differentiation. GSK3 has been shown to be important for T cell-dependent B cell responses (33). Lee et al. found that GSK3 has a regulatory role in GC B cells, especially in the differentiation of GC B cells that have undergone positive selection (34). Additionally, Lee’s findings indicate that the inactivation of GSK results in an elevation of FoxO1 levels. They hypothesize that the augmented cellular concentrations of FoxO1 by GSK3 are attributable to its relocation to the nucleus. This surge in FoxO1 is postulated to be a crucial step in the commencement of the LZ to DZ transition. Lastly, it is noteworthy that the inhibition of GSK3 enhances development into plasma cells and light zone to dark zone transition of GC B cells, indicating that GSK3 has a role in the regulation of B cell differentiation.

When phosphorylated by AKT, The FoxO proteins stop transcription and relocate from the nucleus into the cytoplasm, where they may be degraded by proteolysis. During B cell development, RAG-1 and RAG-2 proteins are needed for the generation of antigen receptors. Amin and colleagues demonstrated that the role of AKT regulate RAG transcription in primary bone marrow B cells and during receptor editing by phosphorylates the FoxO proteins. In pro-B cells and immature B cells, the PI_3_K/AKT signaling axis is key to the regulation of RAG expression (35). Chen et al. determined that the deletion of FoxO1 greatly affects the development of B cells (36). FoxO proteins play a crucial role in class-switch recombination and plasma cell differentiation. AKT inactivates the FoxO proteins to silence those target genes from being expressed, which promotes cell-cycle arrest and apoptosis. Omori et al. proved that the inhibition of class-switch recombination and promotion of antibody-secreting cell differentiation by PI_3_K is mediated by AKT, thus FoxO proteins are phosphorylated by AKT to move out of the nucleus, which causes poor AID expression (37). Collectively, these results demonstrated that FoxO proteins control multiple sets of gene targets that are related to B cell development, peripheral B cell functions, and Ig class switching (21).

## Protein kinase C

**Figure 2:**
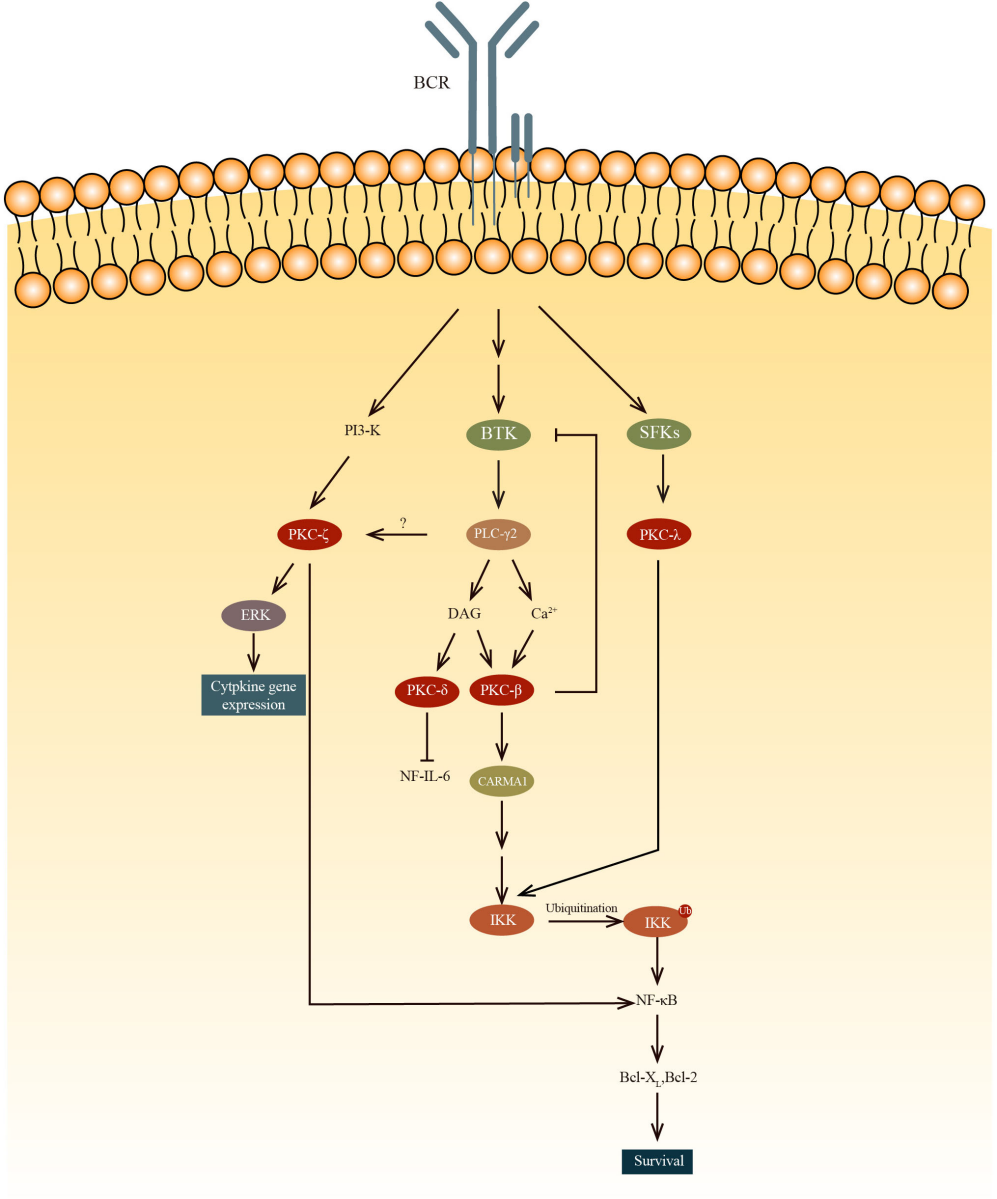
The PKC-β mediated NF-κB activation results in B cell survival while PKC-δ inhibits the NF-IL-6 transcription factor, decreasing IL-6 production and subsequently results in B cell anergy. PKC-ζ phosphorylates RelA, which is an IKK-independent pathway to regulate NF-κB. PKC-ζ was also found to regulate cytokine gene expression by activating ERK. Unlike other PKC, PKCλ can be phosphorylate by SFKs, such as Blk, Fyn and Lyn, ln the PKC-β-NF-κB pathway, IKK is activated by the recruitment and oligomerization of signaling substrates, including Bcl10, which ubiquitinate lKKγ with MALT1 and ubiquitin-conjugating enzyme 13(UBC13).

Protein kinase C is a family of STKs that are critical for cell activation, proliferation, differentiation, and survival (**Figure 2**).

Protein kinase C is divided into two main groups: conventional and novel. Conventional PKCs, including PKCα, PKCβ1, PKCβ2, and PCKγ, require Ca^2+^ and diacylglycerol (DAG) to be activated. Novel PKCs like PKCδ, PKCϵ, PKCη, and PKCθ only require DAG for their activation. There is also a type of PKC that is completely independent of DAG and Ca^2+^ for their activation. Among all these PKCs, PKCα, β1/2, δ, ϵ, η, ζ, and λ are expressed in B-lineage cells (38). It has been demonstrated that PKCs have important roles in the process of development of B cells.

Btk plays a role in PKCβ activation. Btk is involved in BCR-induced NF-κB activation (39–41) and interacts with PKCβ (42). To regulate this activation pathway, activated PKCβ directly phosphorylates Btk to negatively regulate the activity of Btk and its localization in the membrane (43). In PKCβ knockout mice, BCR-dependent cell proliferation and survival are severely impaired because of the deficiency in the induction of two genes(Bcl-2 and Bcl-x_L_) regulated by NK-κB (43, 44). It is revealed that PKCβ knockout mice exhibited a reduction in the number of splenic B cells, a diminished population of B-1 lymphocytes, and diminished levels of serum IgM and IgG3 in comparison to WT mice (45). Fruman and colleagues found that these PKCβ knockout mice had immune disorders similar to individuals with deficiencies in Bruton tyrosine kinase (Btk) or X-linked immunodeficiency (46). However, how the survival of peripheral B cells are regulated by PKCβ through BCR-induced NF-κB is still not clear. Also, the exact role of PKCβ in Btk-mediated activation of NF-κB following B cell receptor (BCR) cross-linking remains to be clarified. Abrams et al. present evidence that the overexpression of PKCβII represents a critical process in the pathogenesis of chronic lymphocytic leukemia. Furthermore, they demonstrate that PKCβII expression levels are negatively correlated with the ability to respond to BCR stimulation (47). In Eμ-PKCβII tg mice, which overexpress PKCβII, there is an expansion of both MZ B cells and B-1 B cells in the spleen and peritoneum, respectively (48). In peripheral blood sample of Eμ-PKCβII tg mice, the immature B cell population also expanded. Furthermore, a significant reduction of BCR signaling and elevated IgM level in serum are also observed in Eμ-PKCβII tg mice. Collectively these results indicated that PKCβ is very important for the survival of B cells.

Miyamoto et al. demonstrated that B cell proliferation is regulated by PKCδ via the regulation of growth-promoting cytokine IL-6 (49). Phosphorylation of NF-IL6 by PKCδ within the DNA-binding domain results in the inhibition of its binding to DNA. This interaction between NF-IL6 and PKCδ reduces expression of IL-6, thereby inhibiting the growth of B cell. Mecklenbrauker and colleagues showed that by participating in the process of B cell anergy, PKCδ plays a role in the proliferation of B cells. As PKCδ is an indispensable component in B cell tolerance signaling, PKCδ deficiency leads to a failure in developing immune tolerance. Self-reactive B cells in PKCδ deficient mice continue to differentiate into APCs, rather than become tolerant. Along with the pro-mitogenic PKCβ, PKCδ is also critical for the fine-tuning of immune responses (50). These two teams have discovered independently that PKCδ controls B cell tolerance (49, 50). They found that splenomegaly and lymphadenopathy are present in PKCδ knockout mice, which can be attributed to the elevated number of peripheral B cells. They also found that the mice died early of severe autoimmune disease, this indicates that PKCδ is important for developing immune tolerance. Additionally, they showed that PKCδ is important for B cell anergy. In PKCδ deficient mice, preventing B cell anergy allows the maturation and differentiation of self-reactive B cells. Collectively, these results indicate that PKCδ regulates B cell proliferation through two distinct pathways, which are inhibition of IL-6 production and control of B cell tolerance.

It is suggested in a study by Saijo et al. that PKCλ may have a role in the NF-κB activation during the early stages of B cell development (51). And unlike any other PKCs, PKCλ is phosphorylated by Src family protein tyrosine kinases (SFKs). Also, the study shows that in the Blk, Fyn, and Lyn triple deficient pro-B cells, the phosphorylation of IKK and activation of NF-κB are significantly reduced. But once paclitaxel, which can activate PKCλ, is added, the defect is restored to normal. To further prove the point that IKK can be activated by PKCλ, a previous study has demonstrated that IKKα and IKKβ are phosphorylated by PKCλ (52).

PKCζ’s role in the immune system is limited to regulating B cell (53). Some studies found that PKCζ directly phosphorylates Ser^311^ of the p65 subunit(RelA) to regulate NF-κB in an IKK-independent pathway (54, 55). In PKCζ deficient mice, B cells exhibit elevated rates of spontaneous apoptosis and demonstrate deficiencies in proliferation and survival in response to IgM cross-linking. Martin et al found that the impaired survival of B cells in PKCζ deficient mice is related to the deficiencies in the extracellular-signal-regulated kinase (ERK) activation and the transcription of genes such as Bcl-xL, and IL-6, the transcription of which are dependent on NF-kB. Furthermore, these genes are inhibited in the IgM-stimulated B cells (in PKCζ deficient mice). Although no major defects in B cell subpopulations was found in PKCζ knockout mice, these mice lost the ability to complete full T-cell-dependent immune response, indicating this defect is a post-B-cell maturation phenomenon.

## Raf kinases and mitogen-activated protein kinases

Raf kinases, which are triggered by the RAS-GTPase, play a role in the MAPK cascade. The Raf kinase family consists of three isoforms: A-Raf, B-Raf, and C-Raf. In B cells, B-Raf and C-Raf are the isoforms that are expressed (56). Raf plays a crucial part in B cell development and function, as the MAPK pathway (RAS-Raf-MEK-ERK) is required for these processes.

In the absence of C-Raf activation in immature B cells, B cell development is significantly impeded, indicating C-Raf has important roles in the early development of B cells. Ititani et al. proved that p21Ras activated C-Raf is important in the development of pro B cells (57). In the past, C-Raf is believed to be the primary transducer in the ERK signaling. Gold demonstrated that the activation of ERK following BCR signaling is mediated by C-Raf (58). At that time the ERK signaling was described as follows: C-Raf, activated by GTP-bound Ras, phosphorylate MEK. MEK in turn phosphorylate ERK, resulting in the transcription of genes like egr-1 or c-fos and the phosphorylation of transcription factors such as Elk-1 and Sap1a. However, this signaling was soon challenged by a series of studies showing that although C-Raf is crucial for prominent ERK activation, it is not the primary activator of ERK signaling (56, 59, 60). Brummer further demonstrated that B-Raf is the primary but not only activator of ERK while C-Raf is only an accessory activator of ERK. In C-Raf deficient DT40 cells, the ERK signaling remained unaltered following BCR stimulation. Conversely, in B-Raf deficient DT40 cells, ERK activation is reduced in both immediate early and late phases the basal ERK phosphorylation is also reduced. As for the induction of egr-1 and c-fos expression, a synergistic effect of B-Raf and C-Raf is also required.

Mitogen-activated protein kinases (MAPK) play a crucial role in the control of cell proliferation, differentiation, and survival. In mammals, MAPKs are classified into three main subfamilies, which consist of ERKs, c-Jun N-terminal kinases (JNKs), and p38 mitogen-activated protein kinases (p38s). In addition to these three subfamilies, ERK3/4, ERK5, and ERK7/8 also have their own unique functions and characters. However, there is little information regarding these MAPK subfamilies, hence are not included in this article.

ERKs are involved in the pre-B cell receptor signaling and are essential for the development of B cells. It has been shown that Erk1 and Erk2 play an indispensable role in the transition of Pro-B cells to Pre-B cells (61). T Yasuda and his team found that ERKs are required in the pre-BCR mediated signaling, including cell expansion mediated by pre-BCR and pro-B cell survival. ERKs are also involved in the fate of B cells. ERKs are activated by C-Raf or PKC and induce proliferation by phosphorylating MYC or cyclin D. Also, strong ERK activation is needed for the development of transitional type 2 B cells into FO B cells (62). The initial phase of differentiation of plasma cells is also dependent on ERK. Two hypotheses were made to explain how ERKs mediate plasma cell differentiation. One is that ERKs induce key transcription factors, such as Blimp-1 (63), and the other is that ERKs play a role in regulating transcription factor activity, abundance, or both in the cytoplasm and nucleus (64). In the former study, it was found that ERKs mainly regulate the expression level of Blimp-1, and Elk-1, which is phosphorylated by ERKs, directly or indirectly increases Blimp-1 transcripts. In T cell dependent immune responses, ERK1 and ERK2 are also indispensable. ERK1 and ERK2 were demonstrated to be essential for the generation of IgG1-secreting plasma cell (64). In TLR signaling pathways ERK1, ERK2, and ERK5 also induce the production of cytokines and chemokines via AP-1 (65). Additionally, ERK is important in inflammation. Carxton and his colleagues showed that BAFF regulated B cell survival is dependent on ERK pathways (66). These studies demonstrate that ERKs are important in the transition of Pro-B to Pre-B cells, differentiation of plasma cells, secretion of IgG1 in plasma cells, inflammation, and B cell survival.

The role of JNK in B cells has not been thoroughly studied. However, it is recognized that JNK is important in cell death (apoptosis, necrosis, and autophagy), cell metabolism, cancer, and the lifespan of a eukaryotic organism (67). It has been demonstrated that JNK is required in the cell death of thymocytes, but its role in B cell death remains unexplored. However, it is confirmed that JNK is essential in the apoptosis of B cells (62). In recent years, JNK signaling has been regarded as a potential autophagy regulation pathway (68). Autophagy is important in activated B cells and for B cell development (69). In B cells, autophagy is induced by BCR signaling (70) and TLR activation (71, 72). The differential regulation of autophagy in activated B cells by BCR engagement and co-stimulation via T cells is thought to create a checkpoint for autoimmunity (73). Autophagy is also suspected to have the ability to promote long-lived plasma cells (71). Several studies have shown that autophagy maintains plasma cell viability over time and also promotes survival in memory B cells and plasma cells (69). In humoral immune responses, autophagy is also critical for the antigen-specific IgM responses against ovalbumin, which is a T cell dependent antigen, in mice (72). This suggests that autophagy is important in humoral immune responses. Given JNK’s potential role in autophagy regulation and the fact that JNK is involved in B cell apoptosis, it is reasonable to suspect that JNKs are very important in B cell development and function. Just like ERKs, JNKs also play the role of promoting the expression of cytokines and chemokines via AP-1 in TLR signaling pathways (65).

p38 mitogen-activated protein kinases (p38 MAPK) signaling pathways are very important in the regulation of inflammation and immune responses. Though p38 MAPK has been confirmed to be very important in the development and function of B cells, its role in the process is still unclear. The p38 MAPK is activated in response to BCR and CD40 stimulation. Khiem D et al. have shown that proliferation of B cells is controlled by the p38 MAPK pathway, which is downstream of the BCR and activated via Mef2c (74). A study by Barrio L et al. has demonstrated that p38γ and p38δ have roles in the B cell’s response to T cell dependent-antigen (75). They also found that in p38γ/δ-/- mice, the spleen size, absolute number of FO B cells, frequency of MZ B cells, and absolute number of MZ B cells were all reduced. P38 signaling pathways affect antibody production by B cells. Norepinephrine increases the level of IgE and IgG1 produced by B cells. G. Pongratz and colleagues have revealed that this process is dependent on the increased expression of CD23, which is dependent on the p38 signaling pathway (76). In plasma cells, p38 also plays an important role in sustaining plasma cell survival (77). The p38 inhibitor, SB203580, prevents the effects induced by IL-17 to promote the survival of plasma cells. Ma’s team further proved that the increase of plasma cell survival is achieved through activated p38 prolonging the half-life of Bcl-xl mRNA. Additionally, p38 promotes cytokine and chemokine expression in TLR signaling pathways via CREB (65).

## Ca^2+^/Calmodulin-dependent protein kinases

Ca2+/Calmodulin-dependent protein kinases (CaMK), are divided into two types: substrate-specific CaMK and multi-functional CaMK. Downstream of CaM, CaMKs are crucial for cells to function properly. CaMKs induce a wide range of cellular events such as gene transcription, cell survival, apoptosis, and cytoskeletal reorganization (78).

Back in the 1990s, Mosialos et al. found that EBV-transformed B lymphoblastoid cell lines express CaMK-Gr, which is abundant in neurons and T cells but absent in B cells. They suggested that CaMK-Gr is a potential mediator of B cell growth transformation (79). B cell activating factor (BAFF) is critically important for B cell survival. Treatment with human soluble BAFF (hsBAFF) promote B cell proliferation and survival. Ke et al. demonstrate that this treatment with hsBAFF leads to extracellular Ca^2+^ influx and ER Ca^2+^ release. The elevated intracellular level of Ca^2+^ elicits phosphorylation of CaMK II thus activating AKT/mTOR signaling (80). Liang and colleagues proved that BAFF-promotes B cell survival and proliferation are dependent on CaMK II inhibition of PP2A (81). BAFF inhibition of autophagy is also activated by CaMK II-dependent AKT/mTOR signaling pathway (82). After AKT/mTOR signaling activation by CaMK II, ULK1 is phosphorylated by mTORC1, which leads to a reduction in the activity of ULK1 kinase. This inhibition of ULK1 kinase results in obstruction autophagosome formation of and inhibition of autophagy. Inhibition of this signaling pathway with mTORC1/2 or AKT inhibitor leads to a reverse of both p-ULK1 increase and reduction in LC3-II, which correlates with the autophagosome formation. Furthermore, inhibition or silencing of CaMK II results in attenuation of the effect of hsBAFF. This finding suggests that CaMK II is an important regulator of B cell autophagy. As CaMK II is a universal integrator of calcium signaling and can rapidly be activated by the increase of intracellular free calcium, the activated CaMK II has the potential to phosphorylate and regulate DNA-binding proteins as well (83). The collective findings suggest that CaMKs play a pivotal role in regulating B cell growth, transformation, survival, proliferation, and autophagy.

## Interleukin-1 receptor associated kinase

Interleukin-1 receptor associated kinase (IRAK) family has four members in humans: IRAK1, IRAK2, IRAKM, and IRAK4. Among the four members, IRAK1 and IRAK4 have kinase activity (84). In humans, IRAK1, IRAK2, and IRAK4 are expressed in B cells, while human IRAKM is expressed specifically in monocytes and macrophages (84, 85). This IRAKM expression pattern is different in other species, for example, murine IRAKM is also found to express in murine pre-B cell line (70Z/3) (86).

Both IL-1R and TLR signaling pathways activate IRAK1. After activation, IRAK1 recruits MyD88 to form a complex with T 6 (87). Huang et al. revealed that IRAK1 has an important role in the Stat3 mediated IL-10 gene expression and is a transcription regulator that directly regulates IL-10 gene transcription (88, 89). In a study on systemic lupus erythematosus, Jacob et al. observed that IRAK1-deficient mice exhibited lower spleen weight, reduced total splenocyte count, decreased total B cells, and reduced levels of IgM and IgG autoantibodies (90). Another study also showed that in human B cells with IRAK1 deficiency, proliferation is impaired and IL-6 production in response to CpG-B stimulation is reduced (91). These all indicate that IRAK1 plays an important role in the proliferation and function of B cells. Similar to IRAK1, IRAK2 also activates NF-κB via MyD88 and TRAF6 interaction. However, the expression level of IRAK2 is lower in B cells compared to IRAK1. Additionally, IRAK2 also interacts with Mal/TIRAP, which is a different TLR intracellular adaptor (89).

IRAK4 is essential in the NF-κB activation in TLR signaling pathways, but dispensable in BCR signaling (92). However, it is noteworthy that IRAK4 deficient human B cells exhibited deficits in proliferation and diminished IL-6 production after being stimulated with CpG-B (91). In humans with the IRAK4^-/-^ mutation, the proportion of HEp-2-reactive IgG^+^ B cells is reduced and IgA^+^ B cells exhibit reduced self-reactivity compared to mature naïve B cells. Schickel et al. also found that IRAK4 deficiency has an impact on the induction of SHM in both IgG^+^ and IgA^+^ B cells (93). And because TLR signals in B cells have the function of maintaining memory B cells, compromised IRAK4 causes impaired CD27^+^IgM^+^ memory responses (94).

## Conclusions

The roles of STKs in B cells are indispensable, as they are crucial regulators of cell proliferation, differentiation, and death (**Table 1**). The majority of frequently studied serine/threonine-specific protein kinases have unique or abundant roles in B cell.

**Table 1:** The roles of serine/threonine-specific protein kinases in B cells.

Serine/threonine- specific protein kinases		Direct Molecular target	Affected signaling pathways	Roles in B cells
Casein Kinase 2		P65/RelA and AKT	NF-κB, JAK/STAT, PI3K/AKT/mTOR, BCR and Notch2-signaling	Regulator for the development and differentiation of B cells
Protein Kinase A		activation-induced cytidine deaminase	PKA and MAPK/Erk signaling	B cell CSR and SHM
Protein Kinase B		FoxO family transcription factors, GSK3, TSC2	BCR and PI_3_K/AKT signaling pathways	Regulates the development, differentiation, and survival of B cells through multiple targets
Protein Kinase C	PKCβ	Btk	NF-κB and PKC signaling	Important for the survival of B cells
	PKCδ	NF-IL6 transcription factors		Controls the tolerance and proliferation of B cells
	PKCλ	IKKα and IKKβ		Regulates B cell development Important for the survival and function of B cells
	PKCζ	ERK and NF-κB		
Raf Kinase		ERK	BCR and MAPK/Erk Signaling	Indispensable role in theRAS-Raf-MEK-ERK cascade
Mitogen-activated protein kinase	ERK JNK P38 MAPK	MYC, cyclin D and Elk-1 ATF2 and Jun MEF2C and MAPKAPK2	BCR and MAPK/Erk Signaling BCR signaling and Regulation of Apoptosis BCR and p38 MAPK Signaling	Essential for B cell development Essential participant in B cell apoptosis Important participant in B cell development and function
CaMK		Ets-1	BCR signaling	Important for B cell survival, proliferation, and autophagy
IRAK		MyD88, TRAF6, Mal/TIRAP	NF-κB signaling	Maintains memory B cells, influences B cell proliferation and IL-6 secretion

The major roles of STKs in B cell are illustrated in this article. CK2 phosphorylates transcription factors and participates in signaling pathways related to B cell development, making it critical for B cell development and differentiation. PKA influences B cell function by intervening in the SHM and CSR processes by regulating AID activity. Akt plays a crucial role in B cell proliferation, development, and apoptosis through phosphorylating downstream substrates, such as TSC2, GSK3, and FoxO family transcription factors. PKC has indispensable functions in B cell survival, development, and function. Three main substrates of MAPK have unique roles in B cell development, apoptosis, autophagy, and antibody-secretion. Although the role of CaMK in B cells is not thoroughly studied, it is proven to be important for B cell survival, proliferation, and autophagy. Similar to CaMK, the three IRAKs expressed in B cells all have potential roles in B cell development and function.

The roles of STKs in B cell development and function are frequently studied, as their significant meaning in diseases such as Castleman disease, systemic lupus erythematosus, asthma (95), and leukemia, the studies regarding the role of STKs in B cells may provide useful information in immunity, and pathophysiology of diseases relating to B cells and may even provide new pharmaceutical targets for B cell diseases. However, there are still many questions needed to be answered in the role of STKs in B cells. Even among the well-studied STKs such as MAPK, there is still much to be discovered. Further investigation is required to elucidate the role of JNK in B cell death and the involvement of p38 MAPK in B cell development and function. Except for those well-studied STK family proteins such as Akt and MAPK, how other STK family proteins regulate B cell development and function is still unclear or the related studies are outdated, leaving gaps in this field. Although it has been demonstrated that CK2 can regulate BCR and Notch2 signaling, the mechanism of regulating the protein expression by CK2 still needs to be explore. It is well established that cAMP is a key second messenger in B cell development and function, indicating PKA may have additional roles in B cell development and function beyond regulating CSR and SHM. As for PKC, numerous questions remain unanswered. For instance, the precise role of PKCβ in Btk-mediated activation of NF-κB remains unclear. Further studies are required to gain insight into the molecular function of IRAK4, particularly with regard to its role in TLR signaling. Furthermore, as new STKs are discovered, their roles in B cells will be intriguing topics to study.

## References

[1] Weekes CD Hidalgo M. Chapter 8 - targeted therapeutics in cancer treatment. In: Prendergast GC Jaffee EM, editors. Cancer Immunotherapy. Academic Press, Burlington (2007). p. 117–48. doi: 10.1016/B978-012372551-6/50072-9

[2] Harnett MM Katz E Ford CA. Differential signalling during B-cell maturation. Immunol Lett. (2005) 98:33–44. doi: 10.1016/j.imlet.2004.11.002 15790506

[3] Dalporto J. B cell antigen receptor signaling 101. Mol Immunol. (2004) 41:599–613. doi: 10.1016/j.molimm.2004.04.008 15219998

[4] Hathaway GM Traugh JA. Cyclic nucleotide-independent protein kinases from rabbit reticulocytes. Purification of casein kinases. J Biol Chem. (1979) 254:762–8. doi: 10.1016/S0021-9258(17)37871-7 216682

[5] Thornburg W Lindell TJ. Purification of rat liver nuclear protein kinase NII. J Biol Chem. (1977) 252:6660–5. doi: 10.1016/S0021-9258(17)39899-X 893433

[6] Kramerov AA Ljubimov AV. Focus on molecules: Protein kinase CK2. Exp Eye Res. (2012) 101:111–2. doi: 10.1016/j.exer.2010.12.011 PMC3437250 21194531

[7] Litchfield DW. Protein kinase CK2: Structure, regulation and role in cellular decisions of life and death. Biochem J. (2003) 369:1–15. doi: 10.1042/bj20021469 12396231 PMC1223072

[8] Quotti Tubi L Mandato E Canovas Nunes S Arjomand A Zaffino F Manni S. CK2β-regulated signaling controls B cell differentiation and function. Front Immunol. (2023) 13:959138. doi: 10.3389/fimmu.2022.959138 36713383 PMC9874936

[9] Wei H Yang W Hong H Yan Z Qin H Benveniste EN. Protein kinase CK2 regulates B cell development and differentiation. J Immunol. (2021) 207:799–808. doi: 10.4049/jimmunol.2100059 34301844 PMC8323969

[10] Lou DY Dominguez I Toselli P Landesman-Bollag E O’Brien C Seldin DC. The alpha catalytic subunit of protein kinase CK2 is required for mouse embryonic development. Mol Cell Biol. (2008) 28:131–9. doi: 10.1128/MCB.01119-07 PMC2223292 17954558

[11] Buchou T Vernet M Blond O Jensen HH Pointu H Olsen BB. Disruption of the regulatory β subunit of protein kinase CK2 in mice leads to a cell-autonomous defect and early embryonic lethality. Mol Cell Biol. (2003) 23(3):908–915. doi: 10.1128/MCB.23.3.908-915.2003 PMC140710 12529396

[12] Taylor SS Yang J Wu J Haste NM Radzio-Andzelm E Anand G. PKA: A portrait of protein kinase dynamics. Biochim Biophys Acta (BBA) Proteins Proteomics. (2004) 1697:259–69. doi: 10.1016/j.bbapap.2003.11.029 15023366

[13] Pérez-Pérez D Santos-Argumedo L Rodríguez-Alba JC López-Herrera G. Role of protein kinase a activation in the immune system with an emphasis on lipopolysaccharide-responsive and beige-like anchor protein in B cells. IJMS. (2023) 24:3098. doi: 10.3390/ijms24043098 36834508 PMC9962394

[14] Raker VK Becker C Steinbrink K. The cAMP pathway as therapeutic target in autoimmune and inflammatory diseases. Front Immunol. (2016) 7:123. doi: 10.3389/fimmu.2016.00123 27065076 PMC4814577

[15] Muto T Okazaki I Yamada S Tanaka Y Kinoshita K Muramatsu M. Negative regulation of activation-induced cytidine deaminase in B cells. Proc Natl Acad Sci. (2006) 103:2752–7. doi: 10.1073/pnas.0510970103 PMC1413812 16477013

[16] Chaudhuri J Tian M Khuong C Chua K Pinaud E Alt FW. Transcription-targeted DNA deamination by the AID antibody diversification enzyme. Nature. (2003) 422:726–30. doi: 10.1038/nature01574 12692563

[17] Yu K. AID function in somatic hypermutation and class switch recombination. ABBS. (2022) 54:759–66. doi: 10.3724/abbs.2022070 PMC9827813 35975606

[18] Basu U Chaudhuri J Alpert C Dutt S Ranganath S Li G. The AID antibody diversification enzyme is regulated by protein kinase a phosphorylation. Nature. (2005) 438:508–11. doi: 10.1038/nature04255 16251902

[19] Pasqualucci L Kitaura Y Gu H Dalla-Favera R. PKA-mediated phosphorylation regulates the function of activation-induced deaminase (AID) in B cells. Proc Natl Acad Sci. (2006) 103:395–400. doi: 10.1073/pnas.0509969103 16387847 PMC1326186

[20] Calamito M Juntilla MM Thomas M Northrup DL Rathmell J Birnbaum MJ. Akt1 and Akt2 promote peripheral B-cell maturation and survival. Blood. (2010) 115:4043–50. doi: 10.1182/blood-2009-09-241638 PMC2875094 20042722

[21] Manning BD Toker A. AKT/PKB signaling: Navigating the network. Cell. (2017) 169:381–405. doi: 10.1016/j.cell.2017.04.001 28431241 PMC5546324

[22] Jellusova J Rickert RC. The PI3K pathway in B cell metabolism. Crit Rev Biochem Mol Biol. (2016) 51:359–78. doi: 10.1080/10409238.2016.1215288 PMC5139348 27494162

[23] Lazorchak AS Liu D Facchinetti V Di Lorenzo A Sessa WC Schatz DG. Sin1-mTORC2 suppresses rag and il7r gene expression through Akt2 in B cells. Mol Cell. (2010) 39:433–43. doi: 10.1016/j.molcel.2010.07.031 PMC2957800 20705244

[24] Zhang Y Hu T Hua C Gu J Zhang L Hao S. Rictor is required for early B cell development in bone marrow. PloS One. (2014) 9:e103970. doi: 10.1371/journal.pone.0103970 25084011 PMC4119011

[25] Baracho GV Cato MH Zhu Z Jaren OR Hobeika E Reth M. PDK1 regulates B cell differentiation and homeostasis. Proc Natl Acad Sci. (2014) 111:9573–8. doi: 10.1073/pnas.1314562111 PMC4084446 24979759

[26] Venigalla RKC McGuire VA Clarke R Patterson-Kane JC Najafov A Toth R. PDK1 regulates VDJ recombination, cell-cycle exit and survival during B-cell development. EMBO J. (2013) 32:1008–22. doi: 10.1038/emboj.2013.40 PMC3616287 23463102

[27] Wicker LS Boltz RC Matt V Nichols EA Peterson LB Sigal NH. Suppression of B cell activation by cyclosporin a, FK506 and rapamycin. Eur J Immunol. (1990) 20:2277–83. doi: 10.1002/eji.1830201017 1700753

[28] Kay JE Kromwel L Doe SEA Denyer M. Inhibition of T and B lymphocyte proliferation by rapamycin. Immunology. (1991) 72:544–9. PMC1384375 1709916

[29] Aagaard-Tillery KM Jelinek DF. Inhibition of human B lymphocyte cell cycle progression and differentiation by rapamycin. Cell Immunol. (1994) 156:493–507. doi: 10.1006/cimm.1994.1193 7517796

[30] Benhamron S Tirosh B. Direct activation of mTOR in B lymphocytes confers impairment in B-cell maturation andloss of marginal zone B cells. Eur J Immunol. (2011) 41:2390–6. doi: 10.1002/eji.201041336 21674478

[31] Gregory MA Qi Y Hann SR. Phosphorylation by glycogen synthase kinase-3 controls c-myc proteolysis and subnuclear localization. J Biol Chem. (2003) 278:51606–12. doi: 10.1074/jbc.M310722200 14563837

[32] Cato MH Chintalapati SK Yau IW Omori SA Rickert RC. Cyclin D3 is selectively required for proliferative expansion of germinal center B cells. Mol Cell Biol. (2011) 31:127–37. doi: 10.1128/MCB.00650-10 PMC3019862 20956554

[33] Jellusova J Cato MH Apgar JR Ramezani-Rad P Leung CR Chen C. Gsk3 is a metabolic checkpoint regulator in B cells. Nat Immunol. (2017) 18:303–12. doi: 10.1038/ni.3664 PMC5310963 28114292

[34] Lee J Park H Lim J Jin H-S Park Y Jung Y-J. GSK3 restrains germinal center B cells to form plasma cells. J Immunol. (2021) 206:481–93. doi: 10.4049/jimmunol.2000908 33380497

[35] Amin RH Schlissel MS. Foxo1 directly regulates the transcription of recombination-activating genes during B cell development. Nat Immunol. (2008) 9:613–22. doi: 10.1038/ni.1612 PMC2612116 18469817

[36] Chen J Limon JJ Blanc C Peng SL Fruman DA. Foxo1 regulates marginal zone B-cell development. Eur J Immunol. (2010) 40:1890–6. doi: 10.1002/eji.200939817 PMC2926184 20449867

[37] Omori SA Cato MH Anzelon-Mills A Puri KD Shapiro-Shelef M Calame K. Regulation of class-switch recombination and plasma cell differentiation by phosphatidylinositol 3-kinase signaling. Immunity. (2006) 25:545–57. doi: 10.1016/j.immuni.2006.08.015 17000121

[38] Mischak H Kolch W Goodnight J Davidson WF Rapp U Rose-John S. Expression of protein kinase C genes in hemopoietic cells is cell-type- and B cell-differentiation stage specific. J Immunol. (1991) 147:3981–7. doi: 10.4049/jimmunol.147.11.3981 1940380

[39] Petro JB Rahman SMJ Ballard DW Khan WN. Bruton’s tyrosine kinase is required for activation of IkB kinase and nuclear factor kB in response to B cell receptor engagement. J Exp Med. (2000) 191(10):1745–54. doi: 10.1084/jem.191.10.1745 PMC2193161 10811867

[40] Bajpai UD Zhang K Teutsch M Sen R Wortis HH. Bruton’s tyrosine kinase links the B cell receptor to nuclear factor kB activation. J Exp Med. (2000) 191:1735–44. doi: 10.1084/jem.191.10.1735 PMC2193152 10811866

[41] Kawakami Y Kitaura J Hartman SE Lowell CA Siraganian RP Kawakami T. Regulation of protein kinase CβI by two protein-tyrosine kinases, btk and syk. Proc Natl Acad Sci. (2000) 97:7423–8. doi: 10.1073/pnas.120175097 PMC16561 10852954

[42] Kang SW. PKCbeta modulates antigen receptor signaling via regulation of btk membrane localization. EMBO J. (2001) 20:5692–702. doi: 10.1093/emboj/20.20.5692 PMC125669 11598012

[43] Su TT Guo B Kawakami Y Sommer K Chae K Humphries LA. PKC-β controls IκB kinase lipid raft recruitment and activation in response to BCR signaling. Nat Immunol. (2002) 3:780–6. doi: 10.1038/ni823 12118249

[44] Saijo K Mecklenbräuker I Santana A Leitger M Schmedt C Tarakhovsky A. Protein kinase C β controls nuclear factor κB activation in B cells through selective regulation of the IκB kinase α. J Exp Med. (2002) 195:1647–52. doi: 10.1084/jem.20020408 PMC2193563 12070292

[45] Leitges M Schmedt C Guinamard R Davoust J Schaal S Stabel S. Immunodeficiency in protein kinase cbeta-deficient mice. Science. (1996) 273:788–91. doi: 10.1126/science.273.5276.788 8670417

[46] Fruman DA Satterthwaite AB Witte ON. Xid-like phenotypes: A B cell signalosome takes shape. Immunity. (2000) 13:1–3. doi: 10.1016/s1074-7613(00)00002-9 10933389

[47] Abrams ST Lakum T Lin K Jones GM Treweeke AT Farahani M. B-cell receptor signaling in chronic lymphocytic leukemia cells is regulated by overexpressed active protein kinase CβII. Blood. (2006) 109:1193–201. doi: 10.1182/blood-2006-03-012021 17003377

[48] Azar AA Michie AM Tarafdar A Malik N Menon GK Till KJ. A novel transgenic mouse strain expressing PKCβII demonstrates expansion of B1 and marginal zone B cell populations. Sci Rep. (2020) 10:13156. doi: 10.1038/s41598-020-70191-y 32753714 PMC7403146

[49] Miyamoto A Nakayama K Imaki H Hirose S Jiang Y Abe M. Increased proliferation of B cells and auto-immunity in mice lacking protein kinase cd. Nature. (2002) 416:865–9. doi: 10.1038/416865a 11976687

[50] Mecklenbräuker I Saijo K Zheng N-Y Leitges M Tarakhovsky A. Protein kinase cdelta controls self-antigen-induced B-cell tolerance. Nature. (2002) 416:860–5. doi: 10.1038/416860a 11976686

[51] Saijo K Schmedt C Su I Karasuyama H Lowell CA Reth M. Essential role of src-family protein tyrosine kinases in NF-κB activation during B cell development. Nat Immunol. (2003) 4:274–9. doi: 10.1038/ni893 12563261

[52] Lallena M-JE Diaz-Meco MT Bren G Moscat J. Activation of IkappaB kinase beta by protein kinase C isoforms. Mol Cell Biol. (1999) 19:2180–8. doi: 10.1128/MCB.19.3.2180 PMC84010 10022904

[53] Martin P. Role of zetaPKC in B-cell signaling and function. EMBO J. (2002) 21:4049–57. doi: 10.1093/emboj/cdf407 PMC126153 12145205

[54] Duran A Diaz-Meco MT Moscat J. Essential role of RelA Ser311 phosphorylation by zetaPKC in NF-kappaB transcriptional activation. EMBO J. (2003) 22:3910–8. doi: 10.1093/emboj/cdg370 PMC169043 12881425

[55] Savkovic SD Koutsouris A Hecht G. PKC zeta participates in activation of inflammatory response induced by enteropathogenic E. coli. Am J Physiol Cell Physiol. (2003) 285:C512–521. doi: 10.1152/ajpcell.00444.2002 12900386

[56] Brummer T. Inducible gene deletion reveals different roles for B-raf and raf-1 in B-cell antigen receptor signalling. EMBO J. (2002) 21:5611–22. doi: 10.1093/emboj/cdf588 PMC131085 12411479

[57] Iritani BM Forbush KA Farrar MA Perlmutter RM. Control of B cell development by ras-mediated activation of raf. EMBO J. (1997) 16:7019–31. doi: 10.1093/emboj/16.23.7019 PMC1170305 9384581

[58] Compans RW Cooper M Hogle JM Ito Y Koprowski H Melchers F. Intermediary signaling effectors coupling the B-cell receptor to the nucleus. Curr Top Microbiol Immunol. (2000) 245/1:77–134. doi: 10.1007/978-3-642-57066-7_3 10533311

[59] Hüser M Luckett J Chiloeches A Mercer K Iwobi M Giblett S. MEK kinase activity is not necessary for raf-1 function. EMBO J. (2001) 20:1940–51. doi: 10.1093/emboj/20.8.1940 PMC125235 11296227

[60] Mikula M Schreiber M Husak Z Kucerova L Rüth J Wieser R. Embryonic lethality and fetal liver apoptosis in mice lacking the c-raf-1 gene. EMBO J. (2001) 20:1952–62. doi: 10.1093/emboj/20.8.1952 PMC125416 11296228

[61] Yasuda T Sanjo H Pagès G Kawano Y Karasuyama H Pouysségur J. Erk kinases link pre-B cell receptor signaling to transcriptional events required for early B cell expansion. Immunity. (2008) 28:499–508. doi: 10.1016/j.immuni.2008.02.015 18356083

[62] Niiro H Clark EA. Regulation of B-cell fate by antigen-receptor signals. Nat Rev Immunol. (2002) 2:945–56. doi: 10.1038/nri955 12461567

[63] Martins G Calame K. Regulation and functions of blimp-1 in T and B lymphocytes. Annu Rev Immunol. (2008) 26:133–69. doi: 10.1146/annurev.immunol.26.021607.090241 18370921

[64] Yasuda T Kometani K Takahashi N Imai Y Aiba Y Kurosaki T. ERKs induce expression of the transcriptional repressor blimp-1 and subsequent plasma cell differentiation. Sci Signal. (2011) 4:ra25. doi: 10.1126/scisignal.2001592 21505187

[65] Gay NJ Symmons MF Gangloff M Bryant CE. Assembly and localization of toll-like receptor signalling complexes. Nat Rev Immunol. (2014) 14:546–58. doi: 10.1038/nri3713 25060580

[66] Craxton A Draves KE Gruppi A Clark EA. BAFF regulates B cell survival by downregulating the BH3-only family member bim via the ERK pathway. J Exp Med. (2005) 202:1363–74. doi: 10.1084/jem.20051283 PMC2212971 16301744

[67] Weston CR Davis RJ. The JNK signal transduction pathway. Curr Opin Cell Biol. (2007) 19:142–9. doi: 10.1016/j.ceb.2007.02.001 17303404

[68] Zhou Y-Y Li Y Jiang W-Q Zhou L-F. MAPK/JNK signalling: A potential autophagy regulation pathway. Biosci Rep. (2015) 35:e00199. doi: 10.1042/BSR20140141 26182361 PMC4613668

[69] Raza IGA Clarke AJ. B cell metabolism and autophagy in autoimmunity. Front Immunol. (2021) 12:681105. doi: 10.3389/fimmu.2021.681105 34163480 PMC8215381

[70] Arnold J Murera D Arbogast F Fauny J-D Muller S Gros F. Autophagy is dispensable for B-cell development but essential for humoral autoimmune responses. Cell Death Differ. (2016) 23:853–64. doi: 10.1038/cdd.2015.149 PMC4832104 26586568

[71] Pengo N Scolari M Oliva L Milan E Mainoldi F Raimondi A. Plasma cells require autophagy for sustainable immunoglobulin production. Nat Immunol. (2013) 14:298–305. doi: 10.1038/ni.2524 23354484

[72] Martinez-Martin N Maldonado P Gasparrini F Frederico B Aggarwal S Gaya M. A switch from canonical to noncanonical autophagy shapes B cell responses. Science. (2017) 355:641–7. doi: 10.1126/science.aal3908 PMC5805088 28183981

[73] Watanabe K Ichinose S Hayashizaki K Tsubata T. Induction of autophagy by B cell antigen receptor stimulation and its inhibition by costimulation. Biochem Biophys Res Commun. (2008) 374:274–81. doi: 10.1016/j.bbrc.2008.07.013 18625201

[74] Khiem D Cyster JG Schwarz JJ Black BL. A p38 MAPK-MEF2C pathway regulates B-cell proliferation. Proc Natl Acad Sci. (2008) 105:17067–72. doi: 10.1073/pnas.0804868105 PMC2579379 18955699

[75] Barrio L Román-García S Díaz-Mora E Risco A Jiménez-Saiz R Carrasco YR. B cell development and T-dependent antibody response are regulated by p38γ and p38δ. Front Cell Dev Biol. (2020) 8:189. doi: 10.3389/fcell.2020.00189 32266269 PMC7105866

[76] Pongratz G McAlees JW Conrad DH Erbe RS Haas KM Sanders VM. The level of IgE produced by a B cell is regulated by norepinephrine in a p38 MAPK- and CD23-dependent manner. J Immunol. (2006) 177:2926–38. doi: 10.4049/jimmunol.177.5.2926 16920928

[77] Ma K Du W Xiao F Han M Huang E Peng N. IL-17 sustains the plasma cell response via p38-mediated bcl-xL RNA stability in lupus pathogenesis. Cell Mol Immunol. (2021) 18:1739–50. doi: 10.1038/s41423-020-00540-4 PMC8245411 32917979

[78] Swulius MT Waxham MN. Ca2+/calmodulin-dependent protein kinases. Cell Mol Life Sci. (2008) 65:2637–57. doi: 10.1007/s00018-008-8086-2 PMC3617042 18463790

[79] Mosialos G Hanissian SH Jawahar S Vara L Kieff E Chatila TA. A Ca2+/calmodulin-dependent protein kinase, CaM kinase-gr, expressed after transformation of primary human B lymphocytes by epstein-barr virus (EBV) is induced by the EBV oncogene LMP1. J Virol. (1994) 68:1697–705. doi: 10.1128/jvi.68.3.1697-1705.1994 PMC236629 8107230

[80] Ke Z Liang D Zeng Q Ren Q Ma H Gui L. hsBAFF promotes proliferation and survival in cultured B lymphocytes via calcium signaling activation of mTOR pathway. Cytokine. (2013) 62:310–21. doi: 10.1016/j.cyto.2013.03.011 23557796

[81] Liang D Zeng Q Xu Z Zhang H Gui L Xu C. BAFF activates Erk1/2 promoting cell proliferation and survival by Ca2+-CaMKII-dependent inhibition of PP2A in normal and neoplastic B-lymphoid cells. Biochem Pharmacol. (2014) 87:332–43. doi: 10.1016/j.bcp.2013.11.006 PMC3896221 24269630

[82] Dong X Qin J Ma J Zeng Q Zhang H Zhang R. BAFF inhibits autophagy promoting cell proliferation and survival by activating Ca2+-CaMKII-dependent akt/mTOR signaling pathway in normal and neoplastic B-lymphoid cells. Cell Signal. (2019) 53:68–79. doi: 10.1016/j.cellsig.2018.09.012 30244168 PMC6289808

[83] Valentine MA Czernik AJ Rachie N Hidaka H Fisher CL Cambier JC. Anti-immunoglobulin M activates nuclear calcium/calmodulin-dependent protein kinase II in human B lymphocytes. J Exp Med. (1995) 182:1943–9. doi: 10.1084/jem.182.6.1943 PMC2192239 7500040

[84] Janssens S Beyaert R. Functional diversity and regulation of different interleukin-1 receptor-associated kinase (IRAK) family members. Mol Cell. (2003) 11:293–302. doi: 10.1016/s1097-2765(03)00053-4 12620219

[85] Wesche H Gao X Li X Kirschning CJ Stark GR Cao Z. IRAK-M is a novel member of the pelle/interleukin-1 receptor-associated kinase (IRAK) family. J Biol Chem. (1999) 274:19403–10. doi: 10.1074/jbc.274.27.19403 10383454

[86] Rosati O Martin MU. Identification and characterization of murine IRAK-M. Biochem Biophys Res Commun. (2002) 293:1472–7. doi: 10.1016/S0006-291X(02)00411-4 12054681

[87] Medzhitov R Preston-Hurlburt P Kopp E Stadlen A Chen C Ghosh S. MyD88 is an adaptor protein in the hToll/IL-1 receptor family signaling pathways. Mol Cell. (1998) 2:253–8. doi: 10.1016/S1097-2765(00)80136-7 9734363

[88] Huang Y Li T Sane DC Li L. IRAK1 serves as a novel regulator essential for lipopolysaccharide-induced interleukin-10 gene expression. J Biol Chem. (2004) 279:51697–703. doi: 10.1074/jbc.M410369200 15465816

[89] Huang Y Misior A Li L. Novel role and regulation of the interleukin-1 receptor associated kinase (IRAK) family proteins. Cell Mol Immunol. (2005) 2:36–9. 16212909

[90] Jacob CO Zhu J Armstrong DL Yan M Han J Zhou XJ. Identification of *IRAK1* as a risk gene with critical role in the pathogenesis of systemic lupus erythematosus. Proc Natl Acad Sci. (2009) 106:6256–61. doi: 10.1073/pnas.0901181106 PMC2669395 19329491

[91] Chiang EY Yu X Grogan JL. Immune complex-mediated cell activation from systemic lupus erythematosus and rheumatoid arthritis patients elaborate different requirements for IRAK1/4 kinase activity across human cell types. J Immunol. (2011) 186:1279–88. doi: 10.4049/jimmunol.1002821 21160042

[92] Suzuki N Saito T. IRAK-4 – a shared NF-κB activator in innate and acquired immunity. Trends Immunol. (2006) 27:566–72. doi: 10.1016/j.it.2006.10.003 17046325

[93] Schickel J-N Glauzy S Ng Y-S Chamberlain N Massad C Isnardi I. Self-reactive VH4-34–expressing IgG B cells recognize commensal bacteria. J Exp Med. (2017) 214:1991–2003. doi: 10.1084/jem.20160201 28500047 PMC5502416

[94] Weller S Bonnet M Delagreverie H Israel L Chrabieh M Maródi L. IgM+IgD+CD27+ B cells are markedly reduced in IRAK-4–, MyD88-, and TIRAP- but not UNC-93B–deficient patients. Blood. (2012) 120:4992–5001. doi: 10.1182/blood-2012-07-440776 23002119 PMC3525023

[95] Zhang Y. From gene identifications to therapeutic targets for asthma: Focus on great potentials of TSLP, ORMDL3, and GSDMB. Chin Med J Pulm Crit Care Med. (2023) 1(3):139–147. doi: 10.1016/j.pccm.2023.08.001 39171126 PMC11332877

